# The effect of pre-operative carbohydrate loading in femur fracture: a randomized controlled trial

**DOI:** 10.1186/s12891-022-05766-z

**Published:** 2022-08-30

**Authors:** Narendra Kumar Chaudhary, Dev Ram Sunuwar, Rachit Sharma, Mandeep Karki, Mukti Nath Timilsena, Anita Gurung, Sunil Badgami, Devendra Raj Singh, Prabesh Karki, Kailash Kumar Bhandari, Pranil Man Singh Pradhan

**Affiliations:** 1 Nepal Orthopaedic Hospital, Kathmandu, Nepal; 2 Department of Nutrition and Dietetics, Armed Police Force Hospital, Kathmandu, Nepal; 3 School of Human and Health Sciences, University of Huddersfield, Huddersfield, UK; 4 Greentara College of Health Sciences, Lalitpur, Nepal; 5 Department of Community Medicine and Public Health, Institute of Medicine, Tribhuvan University, Kathmandu, Nepal

**Keywords:** Femur fracture, Surgery, Pre-operative carbohydrate loading, Nepal

## Abstract

**Background:**

Femur fracture is a major burden among elderly people, leading patients to be bedridden for a long time in the hospital. The body is more likely to be in a catabolic state as a result of the prolonged fasting period required for surgery, leading to an increase in insulin resistance. Pre-operative carbohydrate loading has been shown to improve postoperative outcomes in several countries. The study aimed to evaluate the effect of pre-operative carbohydrate loading in femur fracture surgery.

**Methods:**

This study was single-center, hospital-based, open-label, parallel-group randomized controlled trial conducted between August 2020 and November 2021. A total of 66 participants, aged 50 years and above having femur fractures planned for surgery were included in this study and assigned to the control (*n* = 33) and study (*n* = 33) groups through computer-generated random numbers. The control group was kept fasting from midnight to the next morning as in existence while the study group was intervened with carbohydrate loading according to the Enhanced Recovery After Surgery (ERAS) protocol. The pre-operative nutritional status was identified and the postoperative outcomes were measured using the Visual Analogue Score (VAS), Cumulative Ambulatory Score (CAS), and Modified Barthel Index (MBI) scoring systems. Statistical analyses were performed using the Chi-square test and the Student’s two-sample t-test to compare the outcomes between the two groups.

**Results:**

All the participants completed the study. There was a significant reduction in the average postoperative pain in the carbohydrate loading group (VAS: 4.8 (SD ± 1.8), 95% CI: 4.7–5.4) as compared to the control group (VAS: 6.1 (SD ± 2.1), 95% CI: 5.3–6.8). The average CAS showed a significant improvement in regaining the mobility function of participants in the study group (CAS: 8.1 (SD ± 2.8), 95% CI: 7.1–9.1) than that of the control group (CAS: 6.8 (SD ± 2.8), 95% CI: 5.8–7.8). The mean MBI score of the participants at the time of discharge from the hospital was higher in the study group (MBI:13.1 (SD ± 2.3), 95% CI: 12.2–13.9) compared to the control group (MBI: 11.8 (SD ± 3.1), 95% CI:10.6–12.9). Similarly, the length of hospital stay after surgery had decreased in the study group than in the control group.

**Conclusions:**

The uptake of carbohydrate loading showed reduced post-operative pain, enhanced functional mobility, and decreased length of hospital stay. This study warrants larger trials to show the effect of pre-operative carbohydrate loading in a clinical setting.

**Trial registration:**

NCT04838366, first registered on 09/042021 (https://clinicaltrials.gov/ct2/show/NCT04838366).

**Supplementary Information:**

The online version contains supplementary material available at 10.1186/s12891-022-05766-z.

## Introduction

Femur fracture leads to significant morbidity and mortality and represents one of the leading causes of hospital stay in older people [1]. The annual incidence of femur fracture ranges from 10 to 21 per 100,000 in the world [2, 3]. The annual crude hip fracture is 129 per 100,000 people aged 50 years in India [4]. It is mostly managed by a surgical procedure that creates stress among both patients and relatives. Moreover, people in older life have multi-morbidities such as type-2 diabetes (15%) [5], hypertension (44.9%) [6], thyroid disorder (15.9%) [7], osteoporosis (37.3%) [8] and hypomagnesaemia (48%) [9]. These types of age-related diseases make the treatment of femur fractures more risky and complicated.

According to the current protocol for surgery of femur fracture, the patients are kept in a fasting state from midnight to avoid the risk of pulmonary aspiration during surgery. This causes physical as well as mental discomfort among patients. This is an old technique that has been carried out for many years. Patients undergo a catabolic state after surgery, which can worsen the stress response and contribute to insulin resistance and hyperglycemia lengthening the recovery period [10]. Prolonged fasting for surgery may create different complications such as distress, confusion, instability, headache, dehydration, electrolyte imbalance, postoperative nausea, and vomiting [11]. The procedure of carbohydrate loading is only one small component of a new concept called Enhanced Recovery After Surgery (ERAS) that allows the patients to consume orally 100 g and 50 g of carbohydrates the night before and two hours before surgery respectively [12]. This modern approach has many advantages over traditional practice. Bisch et al., 2019 have mentioned that the physiological reason to provide the evening dose of carbohydrate beverage is to create glycogen stores in the body and the morning dose is to change the body to a “fed” state [13]. Mean gastric emptying time in the pre-operative carbohydrate loading (study) group and two control groups of elderly people having hip fractures showed no clinically important difference [14]. It justifies the findings of a study that prolonged fasting for surgery is unnecessary [11]. Preoperative carbohydrate loading decreases insulin resistance by up to 50% [15] and improves metabolic functions [16]. In cardiac patients, the use of ERAS protocol results in a 35% reduction in postoperative insulin doses [17]. The preoperative carbohydrate loading reduces the expression of Human leukocyte antigen (HLA)-DR on monocytes which decreases the risk of postoperative infection [18] and reduces the intraoperative core body temperature [19]. In addition, it improves muscle function because of less post-operative protein and nitrogen loss [20], which facilitates wound healing. It improves the recovery rate, hence decreasing the length of postoperative stay at hospital [21], and reduces the treatment cost to 15.2% [22]. Similarly, it is also associated with significantly better well-being [23].

In Europe, ERAS is a novel and scientific approach that is mostly used in general surgery, obstetrics, and gynecology [24]. Nutritional support, on the other hand, is considered less important for patients' fast recovery in most orthopedic programs [25]. In Nepal, the traditional approach of administering anesthesia to the patient in prolonged fasting is still in use. To the best of our knowledge, this study is the first of its kind to assess the efficacy of pre-operative carbohydrate loading for femur fracture. The study aimed to assess the effect of pre-operative carbohydrate loading on the improvement of postoperative pain, functional mobility, and the recovery rate among patients undergoing surgery for femur fracture management.

## Methods

### Study design, sample size, and setting

**Fig. 1:**
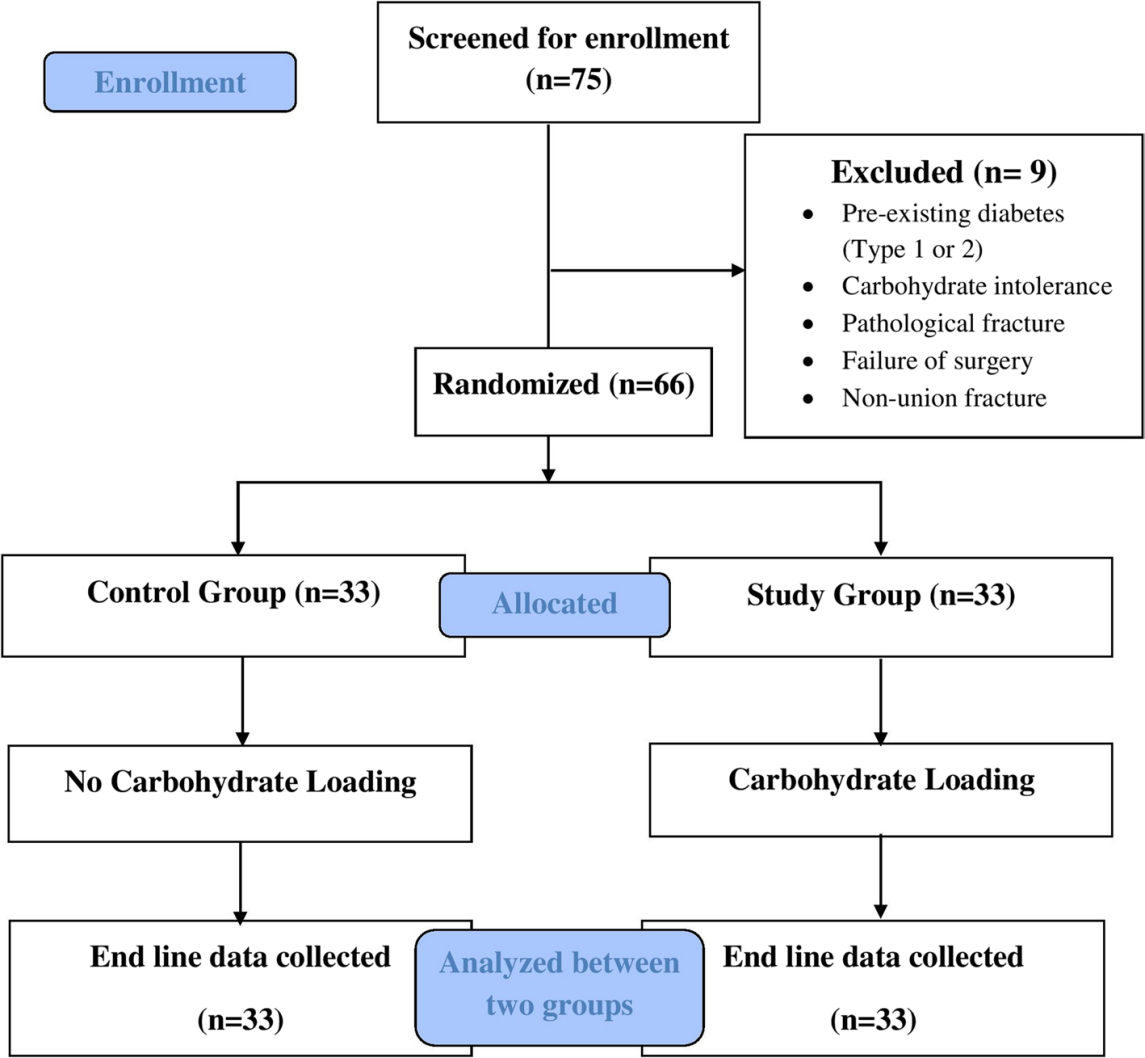
Flow chart of the study

The report of this trial follows the Consolidated Standards of Reporting Trials (CONSORT) 2010 updated guidelines for reporting of randomized controlled trials [26] (Additional file 1). This study was single-center, hospital-based, open-label, parallel-group randomized controlled trial conducted between August 2020 and November 2021 (Fig .1). The study was conducted at the in-patient department of Nepal Orthopaedic Hospital, Kathmandu, Nepal. Nepal Orthopaedic Hospital was selected purposively because it is one of the Orthopaedics and Trauma Care Super-specialty centers in Nepal. According to hospital record, about 10 patients are operated on each day, with one or two cases of femur fracture [27]. The patients aged 50 years and above having a femur fracture planned for surgery, those patients who were mentally fit, and those patients who provided written informed consent were included in the study. Patients with pre-existing diabetes (Type 1 or 2), past carbohydrate intolerance, pathological fracture or any suspected pathology, and surgery failure or non-union cases were excluded from the study. These underlying conditions of the patients were assessed based on medical record and also verified by asking the patient or patient party verbally.

The sample size was determined based on a similar study with the primary outcome being the Cumulative Ambulatory Score (Mean ± SE) of the study group versus the control group as 12.76 ± 0.33 and 12.02 ± 0.32 respectively [23]. Considering this data and taking a level of significance at 5% and power of 80%, the sample size was calculated using a test comparing independent two means in Stata/MP version 14.1 (StataCorp LP, College Station, Texas). The calculated sample size was 60. Taking into account a 10% loss to follow-up and dropout, the total sample size was 66 (33 participants in each group).

### Randomization

Participants were randomized in a 1:1 ratio and assigned to a study and control group randomly. Individual randomization was performed using a computer-generated random number. We created the computer-generated random number using a Microsoft excel sheet and coded control as ‘C’ and study as ‘S’. After that, we prepared envelopes according to a random number, and the participants 1 to 66 were allocated to either control or study group based on the random number. We used an envelope to minimize the selection bias by the researcher. Random numbers were kept in an envelope. Upon confirmation of a participant’s eligibility, the next envelope in the sequence was opened, and the intervention or the control allocation was entered on a randomization list.

### Study variables

#### Outcome variables

The primary outcomes were postoperative pain and functional mobility; serum albumin level was a secondary outcome. The pain on the first postoperative day was assessed during the doctor's round at 10 o’clock in the morning through a visual analog scale (VAS) [28]. It consisted of a 10 cm straight line with the endpoints defining the intensity of pain from zero to 10 indicating that zero as “no pain at all’ and 10 as ‘worst pain’. They were asked to express their severity of pain at the time of data collection in a descriptive term as no pain, mild pain, moderate pain, severe pain, or worst pain, and they were ranked to numerical scores for analysis. Similarly, the Cumulative Ambulatory Score (CAS) was applied to find out the regaining basic mobility independence on the first, second, and third postoperative day like the VAS assessment done at the same time during the doctor's round [29]. It consisted of three aspects of activities; getting in and out of bed, sit-to-stand from a chair, and indoor walking. Each activity was assessed on a three-point ordinal scale from zero to two (0 = “not able”, 1 = “human assistance” and 2 = “independent”), hence a total of daily CAS score ranging from zero to six and zero to 18 for three days assessment [30]. The Modified Barthel Index-Activities of Daily Living (MBI-ADL) was also used to measure independence at the time of discharge from the hospital [31]. The score ranges from 0 to 100; 0–20 indicate “total” dependency, 21–60 indicate “severe” dependency, 61–90 indicate “moderate” dependency and 91–99 indicate “slight” dependency [32]. A score from 0 to 20 for “total dependency” was considered for the study; the higher the score, better the self-care activity.

### Predictor variables

Socio-demographic information of the respondents such as age, sex, Body Mass Index (BMI), occupation, ethnicity, religion, residence area, and educational level were collected using a semi-structured questionnaire interview. Nutritional assessments were done with a Mini Nutritional Assessment Scale (MNA) [33]. It has 18 items related to four different aspects; anthropometric measurement (BMI, weight loss, and arm and calf circumferences); general assessment (lifestyle, medication, mobility, and presence of signs of depression or dementia); short dietary assessment (number of meals, food and fluid intake and autonomy of feeding); and subjective assessment (self-perception of health and nutrition) [34]. They were categorized as having normal nutritional status (if MNA score was 24–30), at risk of malnutrition (if MNA score was 17–23.9), and malnourished ( if MNA score < 17). The clinical parameters such as the site and side of femur fracture, types of fracture, number of fractures, and biochemical parameters like preoperative hemoglobin level and serum albumin level were recorded from the hospital record file.

### Intervention

Having assessed the patients, they were randomized either to the control group or the study group if they met the inclusion criteria. The control group was kept fasting from midnight to the next morning as in existence while the study group was intervened with carbohydrate loading according to the ERAS protocol [35]. We used the glucose-D as a carbohydrate-rich drink of Nepali product (Reg. No.: 3506/045/046, Department of Food Technology and Quality Control, DFTQC No.: 01–33-55–03-218). It contains Dextrose Monohydrate (99.4%), Calcium Phosphate (0.6%) and Vitamin D (0.0001%). The participants were operated on under spinal anesthesia as usual in the hospital setup condition. The intra-operative data regarding the types of surgery, types of implants used, duration of surgery, amount of blood loss, blood transfusion and adverse effect were collected. The hemoglobin and serum albumin level were recorded in the morning of the first post-operative day. The VAS score for pain on the first postoperative day, the CAS score for regaining the mobility function on the first, second and third post-operative days were measured during doctors round at 10 o’clock in the morning. The MBI score was also considered to find out the mobility independence on the day of discharge. Subsequently, all these scoring systems were used to identify the association of pre-operative carbohydrate loading with the recovery rate of patients in terms of the well-being of patients and functional mobility as outcome variables which were further evaluated by the length of hospital stay after surgery.

### Statistical analysis

The data entry was done using EpiData version 3.0, and analyzed based on the intention-to-treat (ITT) approach using Stata/MP version 14.1 (StataCorp LP, College Station, Texas). Descriptive statistics such as frequency, percentage, and mean (standard deviation) were used. Chi-square and Student’s two-sample t-tests were used to compare the outcomes between the study and control groups. All p-values less than 0.05 were considered statistically significant and no adjustment was made for multiple testing.

### Ethical consideration

The ethical approval was obtained from the Ethical Review Board (ERB) of the Nepal Health Research Council (NHRC) (Reg. Number: 3104, approved on 11/05/2021). We also took permission to conduct the research from Nepal Orthopaedic Hospital, Kathmandu, Nepal. We constituted a Data and Safety Monitoring Board (DSMB) consisting of an orthopedic surgeon, dietitian, and statistician. The DSMB members prepared study-stopping rules and reviewed all the possible effects reported in the study. The respondents were informed about the purpose of this study. After obtaining written informed consent, they were recruited as eligible candidates for the study. Voluntary informed participation and freedom of refusal at any time during the study were strongly applied without giving reason and fear. Privacy and confidentiality of the collected information were ensured at all levels. The purpose of the study, benefits, and harm to the participants were properly explained in simple and understandable terms by the team members of the research. No financial benefits or burdens concerned with the study were provided to the patients.

## Results

### Socio-demographic characteristics of the participants

More than half participants were female in the study group while there were more male participants in the control group. The study group had slightly more participants as literate, however, both groups had nearly half of the participants unemployed. Similarly, more participants were from the mountain region in the study group while participants from the Terai region were higher in the control group. **(**Table1**)**.

**Table 1:** Socio-demographic characteristics of participants

Variables	Control group (*n* = 33)	Study group (*n* = 33)
	**n (%)**	**n (%)**
**Sex** Female Male	16 (45.7) 17 (54.8)	19 (54.3) 14 (45.2)
**Age categories (years)** 50–70 71–96	15 (41.7) 18 (60.0)	21 (58.3) 12 (40.0)
**Age in years (mean ± SD)**	69.3 ± 13.9	66.4 ± 11.8
**Education** Literate Illiterate	7 (35.0) 26 (56.5)	13 (65.0) 20 (43.5)
**Ethnicity** Advantaged ethnic group Disadvantaged ethnic group	18 (58.1) 15 (42.8)	13 (41.9) 20 (57.1)
**Religion** Hindu Non Hindu	27 (57.5) 6 (31.6)	20 (42.5) 13 (68.4)
**Occupation** Employed Unemployed	4 (57.1) 29 (49.1)	3 (42.9) 30 (50.9)
**Ecological region** Hill Mountain Terai	12 (54.5) 12 (40.0) 9 (64.3)	10 (45.5) 18 (60.0) 5 (35.7)
**Place of residence** Rural Urban	17 (42.5) 16 (61.5)	23 (57.5) 10 (38.4)

### Clinical assessment of the participants

Both groups had comparable types of femur fractures; proximal femur fracture (neck of femur fracture, inter-trochanteric fracture, and sub-trochanteric fracture), the shaft of femur fracture, and distal femur fracture. Similarly, both groups had a similar pattern of clinical characteristics such as fracture side (left or right), the number of bone fractures, types of implants used, types of surgery performed (Open reduction and internal fixation/Closed reduction and internal fixation), blood loss, blood transfusion, adverse effect, and duration of surgery. However, there was a difference in the pre-nutritional status of the participants. The control group had more participants having normal nutritional status than that of the study group but no difference in malnutrition and risk of malnutrition **(**Table 2**)**.

**Table 2:** Clinical parameters of participants

Variables	Control group	Study group
	**n (%)**	**n (%)**
**Fracture site** Distal femur Proximal femur Shaft of femur	4 (50.0) 20 (47.6) 9 (56.3)	4 (50.0) 22 (52.4) 7 (43.7)
**Fracture side** Left Right	15 (42.8) 18 (58.1)	20 (57.2) 13 (41.9)
**Number of fractures** Two or more Single	4 (57.1) 29 (49.2)	3 (42.9) 30 (50.8)
**Type of surgery done** Open reduction Closed reduction	30 (51.7) 3 (37.5)	28 (48.3) 5 (62.5)
**Type of implants used** Nailing Others Plating	9 (50.0) 1 (100) 23 (48.9)	9 (50.0) 0 24 (51.1)
**Surgery duration** Less than one hour More than one hour	4 (80.0) 29 (47.5)	1 (20.0) 32 (52.5)
**Blood loss** Less than 500 ml More than 500 ml	26 (45.6) 7 (77.8)	31 (54.4) 2 (22.2)
**Blood transfusion** No Yes	26 (46.4) 7 (70.0)	30 (53.6) 3 (30.0)
**Adverse effect** No Yes	33 (51.6) 0	31 (48.4) 2 (100)
**Nutritional status** Malnutrition Risk of malnutrition Normal	7 (53.8) 22 (46.8) 4 (66.7)	6 (46.2) 25 (53.2) 2 (33.3)

### Biochemical parameters of the participants

There were comparable pre-nutritional status, pre-operative as well as post-operative hemoglobin and pre-operative serum albumin. However, the control group showed more loss of serum albumin than the study group in surgery **(**Table 3**)**.

**Table 3:** Comparison of biochemical parameters and pre-nutritional status between the control group and study group

Variables	Control group	Study group
	**Mean ± SD**	**Mean ± SD**
Pre-operative hemoglobin level (gm/dL)	11.2 ± 1.1	11.0 ± 1.2
Post-operative hemoglobin level (gm/dL)	9.9 ± 1.2	9.9 ± 0.9
Pre-operative albumin level (gm/dL)	3.3 ± 0.4	3.4 ± 0.3
Post-operative albumin level (gm/dL)	3.1 ± 0.4	3.4 ± 0.5
Pre-nutritional status	20.6 ± 2.9	20.3 ± 2.5

### Primary outcomes after carbohydrate loading

**Fig. 2:**
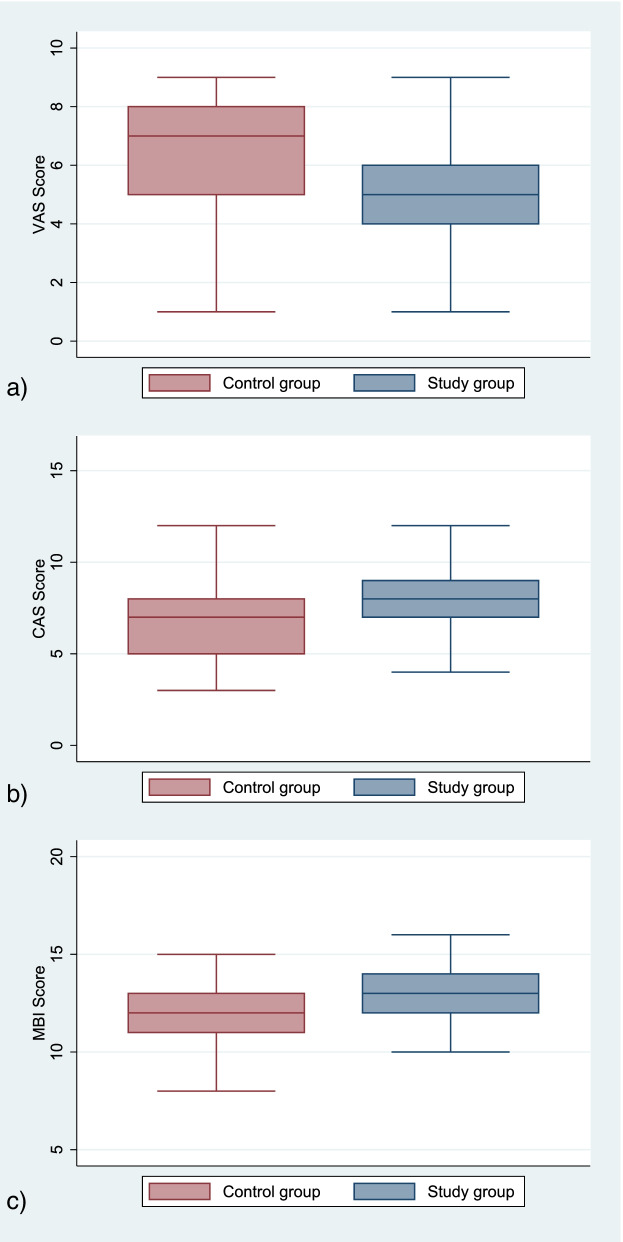
Box plot graph of VAS, CAS, and MBI scores between control and study group

The post-operative pain through VAS was significantly reduced in the study group compared to the control group on the next day of surgery. The cumulative ambulatory score (CAS) showed a significant improvement in regaining the mobility function of participants in the study group than that of the control group. The mean modified Barthel Index (MBI) score of the participants at the time of discharge from the hospital was higher in the study groups. Similarly, the length of hospital stay after surgery was shorter in the study group than in the control group. (Fig. 2 and Table4**)**.

**Table 4:** Comparison of primary outcomes between the control group and study group

Variables	Control group		Study group		*P* value^2^
	**Mean ± SD**	**95% CI**	**Mean ± SD**	**95% CI**	
VAS pain score	6.1 ± 2.1	5.3–6.8	4.8 ± 1.8	4.7–5.4	0.010
CAS score	6.8 ± 2.8	5.8–7.8	8.1 ± 2.8	7.1–9.1	0.033
Length of hospital stay	8.8 ± 4.5	7.2–10.4	6.7 ± 2.4	5.8–7.6	0.024
Modified Barthel Index (MBI)	11.8 ± 3.1	10.6–12.9	13.1 ± 2.3	12.2–13.9	0.027

^2^Student’s two-sample t-test

## Discussion

Pre-operative carbohydrate loading had no adverse effects in our study, which is consistent with prior findings [36, 37]. According to Loodin and Hommel, the carbohydrate loading reduced post-operative complications associated with hip fracture by 50% [38]. In our study, two participants experienced hypoglycemia during surgery, which was managed by the attending anesthesiologist in the operation theater **(**Table 2**)**. A systematic review by Sebranek et al., 2013 showed that the alterations in blood glucose might be multifactorial and other modifiers may be concerned with its homeostasis [39].

Postoperative pain reduction or elimination with minimum side effects is an important quality measure of patients’ well-being and satisfaction that leads to a shorter length of hospital stay and reduced hospital costs [40]. This research showed that pre-operative carbohydrate loading significantly reduced post-operative pain, which is in line with previous findings [23, 41]. The spectrum of fasting induces the C-Reactive Protein (CRP), a marker of subclinical inflammation [42], which is the underlying origin of all pain [43]. The higher the CRP level, the more the oxidative stress [44]. Perrone et al., 2011 found a higher postoperative serum CRP in the control group than that of the preoperative carbohydrate drinks group [45]. Hence, preoperative carbohydrate drinks reduce postoperative oxidative stress, [46] and it is related to the reduction of functional pain [47]. Besides these, it can be explained that dietary intake can promote the function of the nervous system, immune system, and endocrine system which has an impact on pain experience [48].

The regaining of functional mobility after the surgery is a milestone in hospital rehabilitation [49]. We assessed the mobility function with the cumulative ambulatory scores on the 1^st^, 2^nd^, and 3^rd^ postoperative days. The CAS score of the study group was significantly higher than that of the control group. This finding is consistent with the study of Chada et.al in India [23]. The carbohydrate drinks before surgery improve carbohydrate uptake, utilization, storage, and protein metabolism with a 50% reduction in loss of lean body mass [50]. Preoperative carbohydrate beverages help to store glycogen in the muscle [13] and prevent the loss of lower limb mass leading to better postoperative recovery [19].

The MBI scoring system evaluates the activities of daily living (ADL) based on the degree of assistance required to perform the task after a stroke or surgery [51]. Our study found a higher value of MBI-ADL index score in the study group than that of the control group. Ping et, al (2021) also found a dramatically higher Barthel score of self-care ability among the elderly patients with the ERAS approach in total hip replacement surgery [24].

Due to the occurrence of a more catabolic state during the surgery period, there occurs the destruction of many important inorganic as well as organic elements (for example nitrogen and protein loss) in the body, altering the speed of wound healing [52]. One of them is the decrement of serum albumin, commonly observed in most patients [53] which is the result of intense surgical stress predicting increased postoperative complications [54]. The present study found that the preoperative carbohydrate prevented the decrease of serum albumin in the body during surgery, consistent with the study [45]. It can be explained through the mechanism that preoperative fasting induces perioperative insulin resistivity which inhibits the synthesis of serum albumin [55]. Serum albumin has several physiological functions and the determined serum albumin reflects the physical or health status, predicting the prognosis [56]. Low serum albumin leads to a poor prognosis [57]. It is the marker for surgical stress that may delay the clinical outcome [58] and occur the mortality of patients [59].

The reduction of hospital stay facilitates financial, operational, and clinical outcomes [60]. This study reveals the decrease in hospital stay with carbohydrate loading supported by different studies [21, 22, 61–63]. Amer et. al find that pre-operative carbohydrate drinks reduce the length of postoperative stay at the hospital from 0.4 to 0.2 days compared with fasting [64]. Kotfish et al., 2020 find a 50% reduction in the length of ICU stay after cardiac surgery [17].

Our study has a few limitations. We could not evaluate the insulin resistivity and other hematological parameters due to the high cost and also the unavailability of most of the investigations in the laboratory of the study center. Furthermore, the patient's medical co-morbidities based on the American Society of Anesthesiologists (ASA) could not be analyzed to rule out the severity of health [65], which affects the recovery rate. The sample size for this study was limited and the data were collected at the time of the first wave of the COVID-19 pandemic in Nepal which interrupted the data collection process.

## Conclusion

Pre-operative carbohydrate loading in femur fractures accelerates recovery rate in terms of postoperative pain and ambulatory function, shortening hospital stay. This study provides preliminary evidence on the benefits of pre-operative carbohydrate loading and warrants larger trials with a higher sample size for stronger evidence. Further research can be conducted using beverages that contain carbohydrates, fat, protein, and other micronutrients to acquire additional better postoperative outcomes.

## Supplementary Information

**Additional file 1.** CONSORT 2010 checklist of information to include when reporting a randomised trial*.

## Data Availability

The data that supports the findings of this study are available on request from the corresponding author. The data are not publicly available due to privacy or ethical restriction.

## Abbreviations

ADL: Activities of Daily Living
ASA: American Society of Anesthesiologists
BMI: Body Mass Index
CAS: Cumulative Ambulatory Score
CRP: C-Reactive Protein
DSMB: Data and Safety Monitoring Board
ERAS: Enhanced Recovery After Surgery
ERB: Ethical Review Board
VAS: Visual Analogue Score
MBI: Modified Barthel Index
MNA: Mini Nutritional Assessment
NHRC: Nepal Health Research Council
